# Revisiting CPSF30-mediated alternative polyadenylation in *Arabidopsis thaliana*

**DOI:** 10.1371/journal.pone.0319180

**Published:** 2025-02-24

**Authors:** Guijie Hao, Lichun Zhou, Huazhen Liu, Pradeep Kachroo, Arthur G. Hunt

**Affiliations:** 1 Department of Plant and Soil Sciences, University of Kentucky, Lexington, Kentucky, United States of America; 2 Department of Plant Pathology, University of Kentucky, Lexington, Kentucky, United States of America; Centre de Recherche en Biologie cellulaire de Montpellier, FRANCE

## Abstract

Alternative polyadenylation (APA) is an important contributor to the regulation of gene expression in plants. One subunit of the complex that cleaves and polyadenylates mRNAs in the nucleus, CPSF30 (for the 30 kD subunit of the mammalian Cleavage and Polyadenylation Specificity Factor), has been implicated in a wide-ranging network of regulatory events. CPSF30 plays roles in root development, flowering time, and response to biotic and abiotic stresses. CPSF30 also is a conduit that links cellular signaling and RNA modification with alternative RNA processing events and transcriptional dynamics. While much is known about CPSF30 and its roles in plants, questions remain regarding the connections between CPSF30-mediated APA and the downstream events that lead to specific phenotypic outcomes. To address these, we conducted a detailed analysis of poly(A) site usage in the CPSF30 mutant. Our results corroborate earlier reports that link CPSF30 with a distinctive cis element (AAUAAA) that is present 10-30 nts upstream of some, but not all, plant pre-mRNAs. Interestingly, our results reveal a distinctive shift in poly(A) site in mutants deficient in CPSF30, resulting in cleavage and polyadenylation at the location of motifs similar to AAUAAA. Importantly, CPSF30-associated APA had at best a small impact on mRNA functionality. These results necessitate the formulation of new hypotheses for mechanisms by which CPSF30-mediated APA influences physiological processes.

## Introduction

Alternative polyadenylation (APA), the process wherein a primary transcript may be cleaved and polyadenylated at different sites, is a crucial regulator of gene expression in eukaryotes. In mammals, APA is important for growth and development and is an important factor in diseases such as cancer [1–5]. In plants, APA affects growth and development as well as the responses of plants to environmental signals such as abiotic and biotic stresses [6–8]. Messenger RNA polyadenylation is mediated by a large complex; many subunits of this complex have been linked with APA in several physiological and developmental contexts (e.g., [9–28],). One subunit, CPSF30 (for the 30 kD subunit of the mammalian Cleavage and Polyadenylation Specificity Factor), has been implicated in numerous processes [9,25,26,29–36] and is a bridge between cellular signaling, RNA modification, and APA [32,36,37]. In contrast to mammals and yeast, plants possess two CPSF30 isoforms, CPSF30S and CPSF30L, that are encoded by a single gene and produced via alternative processing of the primary transcript [25,38,39]. CPSF30S corresponds to the CPSF30 protein found in mammalian and yeast and consists of a core of three CCCH zinc finger motifs that interact with RNA, other subunits of the polyadenylation complex, and calmodulin [25,29,37,39–43]. CPSF30L possesses all of the functionality of CPSF30S but also includes an additional domain that acts as a reader of m6A modifications in RNA [32,36].

In mammals and yeast, CPSF30 is an essential protein [44–46]. In contrast, in plants, CPSF30 seems to be dispensable since plants with null mutations in the gene that encodes CPSF30 (AT1G30460) are viable [25,26,34]. Mutations in *Arabidopsis* that alter or abrogate CPSF30 expression have wide-ranging effects on poly(A) site choice [23,32,36,47] as well as transcription termination [35]. These effects are thought to be the basis for the many phenotypes seen in CPSF30-deficient plants, although clear causal relationships between CPSF30-dependent APA and specific phenotypes have not been definitively established. Additionally, the mechanism(s) underlying the shifts in poly(A) site usage observed in CPSF30-deficient plants remains poorly understood.

In this report, we revisit the control of poly(A) site selection by CPSF30 in *Arabidopsis*. through a detailed meta-analysis of existing datasets and new poly(A) site profiling. We find that CPSF30-dependent sites exhibit a strong preference for AAUAAA-containing polyadenylation signals, corroborating prior reports linking the *Arabidopsis* CPSF30 with AAUAAA-like motifs [23,47]. We also show that a loss of CPSF30 leads to the unmasking of a set of poly(A) sites with a distinctive sequence profile, characterized by a strong preference for U-rich polyadenylation signals and the presence of sequences related to AAUAAA at the cleavage site. We also find that the vast majority of poly(A) sites whose usage is influenced by CPSF30 lie within 50 nucleotides of another poly(A) site, with most being closer than 20 nucleotides. These intervals do not overlap with known microRNA target sites and are likely too small to have a significant impact on mRNA function. These results raise interesting questions regarding the processing and polyadenylation of mRNA precursors in plants and indicate that the global remodeling of poly(A) site usage in CPSF30-deficient mutants may not substantially affect mRNA function in most instances.

## Materials and methods

### Growth of plants and RNA isolation

For the two new independent sets of sequencing data used for the determination of CPSF30-dependent APA, wild-type *Arabidopsis* (ecotype Col-0) and the *cpsf30-1* mutant (also known as *oxt6* [25,26];) were used. For one study, seed were surface-sterilized by incubating in 70% ethanol for 1 min, followed by treatment with 10% bleach for 10 min, and then rinsed with distilled water five times. After sterilization, seeds were suspended in 0.1% agar solution and sewn onto media containing half-strength Murashige and Skoog salts, 1% (w/v) sucrose, and 0.8% (w/v) agar, all at pH 5.7. Following stratification for 2 d in the dark at 4°C, plates were transferred to a growth chamber under long-day conditions (16 hr light) at 22 °C for 3 weeks, after which time they were removed and frozen in liquid nitrogen. All seedlings were collected at the same time of day, to minimize the contribution of circadian effects to possible differences in poly(A) site choice [48]. Frozen seedlings were stored at -80°C until used for RNA isolation.

For the other study, plants were grown in MTPS 144 or MTR-30 Conviron (Winnipeg, MB, Canada) walk-in or reach-in chambers set at 22 °C, 65% relative humidity. These chambers were equipped with cool white fluorescent bulbs (Sylvania, FO96/841/XP/ECO). The photon flux density (PFD) of the day period ranged from 120 µmoles m^-2^ s^-1^ (measured using a digital light meter, Phytotronic Inc, MO and the chamber microprocessor). The chambers were set at 12 h light. Plants were grown on autoclaved Pro-Mix soil (Premier Horticulture Inc., PA, USA). Soil was fertilized once using Scotts Peter’s 20:10:20 peat lite special general fertilizer that contained 8.1% ammoniacal nitrogen and 11.9% nitrate nitrogen (Scottspro.com). Plants were irrigated using deionized or tap water. Plants were sampled directly (without storage) for RNA isolation as described below. All samples were collected at the same time of day, to minimize the contribution of circadian effects to possible differences in poly(A) site choice [48].

### Determination of mRNA stability

Col-0 and *cpsf30-1* seeds were sterilized (see above) and sowed on solid media (1x Murashige and Skoog salts, MES 0.5g/L pH =  5.7, 1% (w/v) sucrose, 0.8% (w/v) agar). Plates were incubated in vertical orientation in the dark at 4°C for two days and then shifted to room temperature under long day conditions (16h light and 8h dark). After two weeks, whole seedlings were transferred to a Petri dish containing incubation buffer (15 mM sucrose, 1 mM KCl/ 1 mM Pipes/ 1 mM sodium citrate, pH 6.5) and incubated for 30 min at room temperature before addition of cordycepin (200 µ M). Vacuum was applied for 30 seconds, and plants then incubated for an additional 60 min. Controls were treated identically, but cordycepin was not added. Plants were harvested after 60 min and were immediately frozen in liquid nitrogen. In all experiments, three biological replicates were prepared.

### RNA isolation, PATSeq library preparation, and data analysis

Frozen or fresh plant tissue was ground with a mortar and pestle and RNA extracted using TRIzol RNA Isolation Reagents (Life Technologies) as recommended by the manufacturer. RNA quality and concentration were determined by gel electrophoresis and determination of A_260_. Short read sequencing libraries that query the mRNA-poly(A) junction (PAT-Seq libraries) were subsequently prepared using this isolated RNA as described previously [31,49–51]. Libraries were sequenced at the University of Kentucky Healthcare Genomics Core facility. In addition to the sequencing data generated for this study, an additional dataset described in Yu et al. [23] was downloaded. These four datasets—three wt-mutant comparisons and the results of the stability experiment—were unpacked, demultiplexed, and further analyzed using the pipelines described previously [31,51–54], with one important exception. Specifically, in the analyses conducted in this report, poly(A) sites that were near each other were not grouped into larger clusters but were treated as individual sites for the purposes of further characterization. Motif analysis was conducted using the MEME-ChIP package [55] on the Galaxy platform [56]. The most prominent A-rich and U-rich motifs were further analyzed and visualized using Excel. Proximities of different poly(A) sites was determined using the closestbed tool in the Bedtools suite [57]. MicroRNA target sites were from the TarDB database [58]. Gene Ontology analysis was conducted using the ShinyGO package [59]. Boxplots were generated using BoxPlotR [60]. In the box plots, notches in the boxes show the approximate 95% confidence interval by which the hypothesis that two medians differ can be evaluated [60].

## Results

### Description of datasets used in this analysis

The *Arabidopsis* CPSF30 protein has been associated with control of poly(A) site choice and insights into the scope of CPSF30-associated APA have been reported [23,32,35,36,47]. However, questions remain concerning the plant CPSF30 and its functioning in mRNA polyadenylation. To address some of these, we chose to identify and characterize high-confidence CPSF30-dependent poly(A) sites in *Arabidopsis*. For this, we aimed to access or generate independent datasets for subsequent analysis. Our rationale was that commonalities observed across multiple experiments and datasets are more likely to reflect genuine associations between CPSF30 and changes in poly(A) site selection. To this end, we utilized available PATSeq data [23] that included the wild type Columbia (Col-0) ecotype and the *oxt6* mutant (also referred to as *cpsf30-1*) and generated two new datasets derived from seedlings grown on defined media or soil-grown plants. All of these data were generated using the same library preparation strategy and the datasets had comparable numbers of total and mapped reads (S1 File), allowing for both comparison and pooling in subsequent studies.

**Fig 1:**
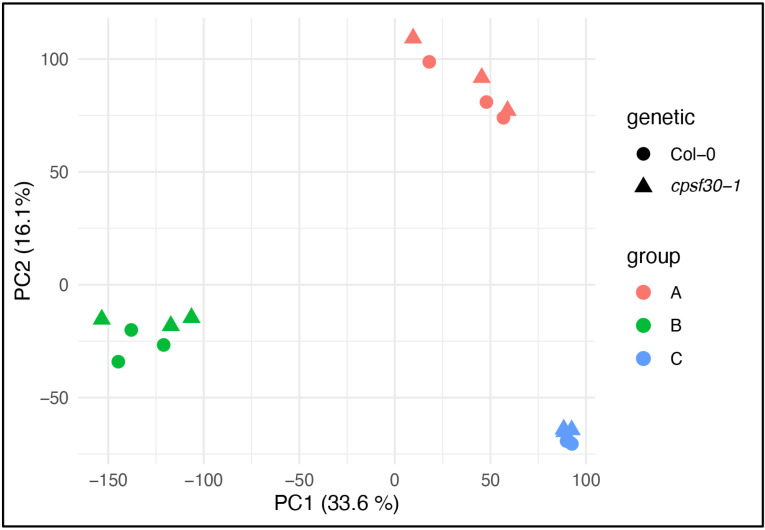
PCA analysis of the three experiments used to derive the set of CPSF30-dependent and *oxt6-*specific poly(A) sites. Gene expression was determined for each of the 18 samples used to study CPSF30-dependent poly(A) site choice. Wild-type (Col-0) and mutant (*cpsf30-1)* samples are denoted with circles and triangles, respectively. The three experiments are color-coded as shown in the figure. Group A are plants grown on defined media, Group B are soil-grown plants, and Group C are derived from the data from Yu et al. [23]. The data used to generate these plots are provided in S2 File.

To evaluate the impact of different conditions on expression dynamics, we conducted an overall assessment of gene expression. Principal component analysis (PCA) of the genome-wide gene expression under various conditions showed consistent clustering of replicates (Col-0 and *cpsf30-1*) across all three studies. The experiment-specific differences were more pronounced than any discernible differences between the Col-0 and *cpsf30-1* plants (Fig 1). This finding informed our subsequent analyses of poly(A) site usage. We reasoned that while there may be genuine or potentially significant differences between mutant and wild-type lines that will depend on specific growth conditions, these differences could be difficult to distinguish from those caused by inherent experimental variability. Although it may be possible to identify these differences, we chose to set these aside and focused on differences consistently observed in all datasets as these are likely to reflect the true effects of CPSF30 deficiency.

The rationale behind this study is that different classes of poly(A) sites will shed light on CPSF30-mediated APA. Specifically, there should be sites that are present in the wild-type but absent in CPSF30-deficient mutants. The characteristics of these sites will provide insights into the sequences specifically recognized by CPSF30. Additionally, there should be sites that only appear in the *cpsf30-1* mutant. These sites will reflect the activity of a polyadenylation complex lacking CPSF30, offering clues to the mechanism by which such complexes function. Finally, there should be a set of sites that are present in both wild-type and mutant lines. This class of sites would highlight features of the polyadenylation complex that function independently of the of CPSF30.

In previous analyses of APA in CPSF30-deficient *Arabidopsis* mutants, individual poly(A) sites were typically grouped into clusters. This was not done in the study presented in this report; rather, we assumed that there may be information in individual sites that prior studies may have missed. Accordingly, the following results describe the properties of individual sites and not clusters.

### Characteristics of CPSF30-dependent poly(A) sites

To identify poly(A) sites that rely on CPSF30 for their usage, sites were identified that had significantly different usage between Col-0 and *cpsf30-1* plants (p-value of a two-sided Student’s t-test < 0.01) and with a fractional usage at least ten-fold higher in the Col-0 plants compared to the *cpsf30-1* mutant. This analysis yielded a set of 2428 CPSF30-dependent poly(A) sites (S3 File), representing 1268 genes. The discrepancy between the numbers of sites and genes reflects the tendency for polyadenylation to occur within small clusters of sites.

**Fig 2:**
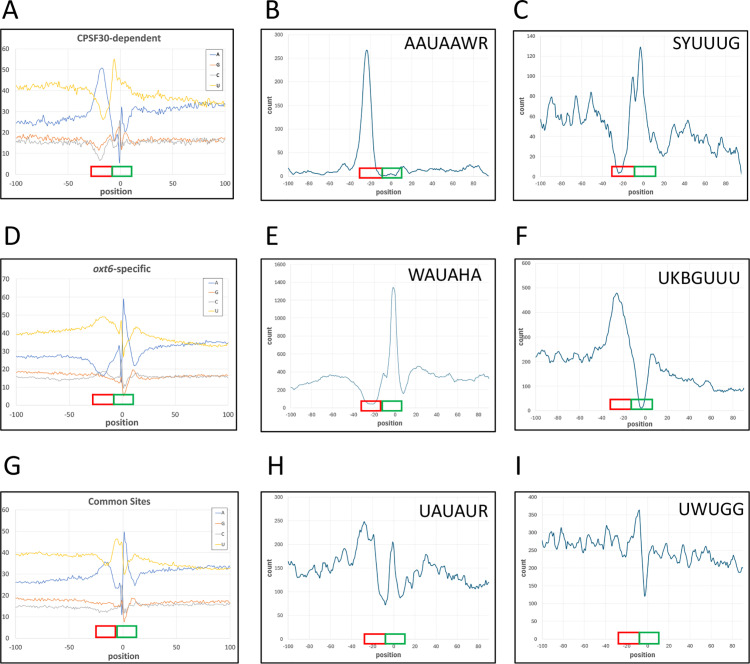
Characteristics of different classes of poly(A) sites. Panels A, D, and G show the average nucleotide compositions surrounding CPSF30-dependent (panel A), *oxt6-*specific (D), and common (G) sites. X-axes on these denote positions relative to the poly(A) site (defined as position 0). Y-axes denote the percent composition for each base at each position. Panels B, E, and H show the normalized frequencies of occurrence of the most frequent AAUAAA-related motifs relative to CPSF30-dependent (panel B), *oxt6-*specific (E), and common (H) sites. Panels C, F, and I show the normalized frequencies of occurrence of the most frequent U-rich motifs relative to CPSF30-dependent (panel C), *oxt6-*specific (F), and common (I) sites. For panels B, C, E, F, H, and I, X-axes denote the position, relative to the poly(A) site, of the first base of the 6 nt motif. For these panels, Y-axes show absolute counts for each class of motif. In these panels, the specific motif that was mapped is indicated at the top tight of the plot. In all nine panels, the locations of Near-Upstream Elements (NUEs) and Cleavage Elements/sites (CE) are represented with red (NUE) and green (CE) boxes above the X axes. The five most-frequent motifs for each class of site are shown in S1 Fig.

The nucleotide composition around these was measured and plotted (Fig 2A). The results show that CPSF30-dependent sites are characterized by a pronounced A-rich region 10-30 nts upstream of the poly(A) site (Fig 2A), corresponding to the Near Upstream Element (NUE) [61–63]. These results agree with previous observations [23,47]. The region between 1-10 nts upstream of the poly(A) site is notably U-rich, a trend that continues, though less prominently, to the 10 nts downstream of the poly(A) site (Fig 2A). Motif analyses revealed a clustering of AAUAAA-like sequences in the NUE region (Fig 2B), consistent with its overall A-rich nature. Additionally, U-rich motifs cluster around the cleavage site itself (Fig 2C), reflecting the strong U-rich composition in this region (Fig 2A).

### Characteristics of poly(A) sites observed only in the *cpsf30-1* mutant

Using criteria described in the preceding section, we identified 10,291 poly(A) sites primarily or exclusively utilized in the absence of CPSF30 (S3 File). These are termed in the following and in the figures as *oxt6-*specific, reflecting the original designation (*oxt6*) of the *cpsf30-1* mutant. These sites affected 3512 genes (S3 File).

As was done for the CPSF30-dependent sites, the nucleotide compositions surrounding *oxt6-*specific sites was measured and plotted (Fid. 2D). The results show that *oxt6*-specific poly(A) sites exhibit a pronounced U-rich region 10-30 nts upstream of the poly(A) site (Fig 2D), corresponding to the NUE and in the same position as the A-rich peak found in CPSF30-dependent sites. The region between -10 and + 10 is distinctly A-rich (Fig 2D). This A-rich feature is absent in CPSF30-dependent sites (Fig 2A) but is observed in common sites (discussed in the following subsection; Fig 2G). Further downstream of *oxt6-*specific sites, a notable U-rich region is present (Fig 2D). Motif analysis of *oxt6-*specific poly(A) sites reveal a clustering of AAUAAA-like motifs around or at the position of cleavage and polyadenylation site (Fig 2E), along with clustering of U-rich motifs corresponding to the U-rich region seen in Fig 2C (Fig 2F).

### Characteristics of common poly(A) sites

Using criteria described in the preceding sections, we identified 12,352 poly(A) sites whose usage is independent of the presence or absence of CPSF30 (S3 File). These sites are associated with 5174 genes. This number of sites was somewhat lower than expected based on a previous study [47]. In this earlier report, about 30% of all identified sites were defined as common sites, while we find that about 50% of common sites identified here are common sites. This discrepancy probably reflects the approach towards defining poly(A) sites (clusters vs. individual sites) and the filters used in the present study that required the presence of sites in multiple experiments and at a relatively high expression level compared with the earlier report.

The general nucleotide composition around common sites was similar to that of CPSF30-dependent sites in that there was a noticeable A-rich region at the position typically associated with the NUE (Fig 2G). However, common sites display a distinctive nucleotide composition surrounding the cleavage site, characterized by a U-rich region surrounding an A-rich peak at the cleavage site (Fig 2G). These features appear to be a hybrid of those observed in CPSF30-dependent and -specific sites. Motif analysis revealed a modest enrichment of A + U-related motifs (including AAUAAA) in the NUE and CE regions (Fig 2H). Consistent with the nucleotide composition of the CE region, the distributions of AAUAAA-related motifs also appear to be a combination of those found in CPSF30-dependent and *-*specific sites. There is, at best, a slight enrichment of UG-related motifs immediately upstream of the cleavage site (Fig 2I).

### Genome-wide features of CPSF30-dependent and -independent sites

To gain further insight into the potential roles of CPSF30-dependent polyadenylation, we conducted gene enrichment analyses. These results (S2 Fig) showed that genes with CPSF30-dependent (S2 Fig, panels A-C) or *oxt6*-specific (S2 Fig, panels D-F) sites were enriched for functions related to organelle and ribosome function, among other categories. Genes with multiple poly(A) sites none of which were strain-specific showed no significant enrichment for any particular GO classification. Genes with single poly(A) sites were significantly enriched for functions associated with transcriptional regulation (S2 Fig, panels G and H and legend).

**Fig 3:**
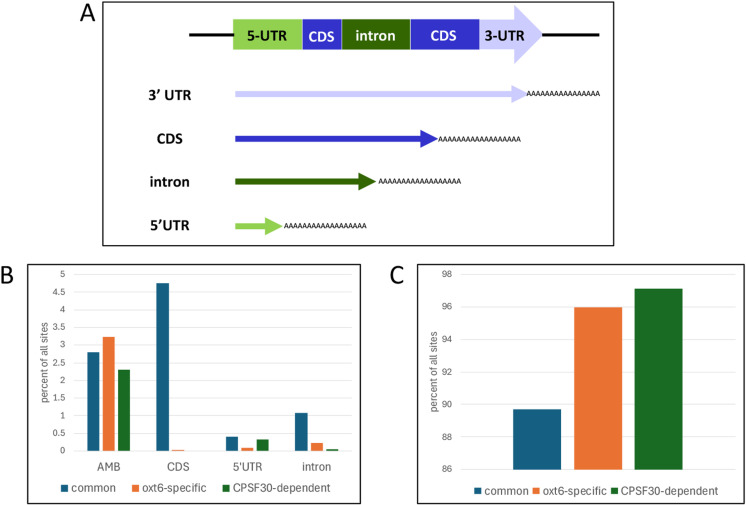
Genomic locations of common poly(A) sites, *oxt6-*specific sites, and CPSF30-dependent sites. Panel A illustrates the definitions of the locations. Panel B shows the distribution of sites in locations apart from 3’-UTRs. Panel C illustrates the distribution of sites that lie within 3’-UTRs. AMB denotes sites that fall within regions that have multiple annotations.

Poly(A) sites may be situated at different positions within a gene, including the 3’-UTR, introns, protein-coding regions, and 5’-UTRs [64]. The location of the poly(A) site can have regulatory significance. Accordingly, we analyzed the genomic locations of common, CPSF30-dependent, and *oxt6-*specific sites. The results show that CPSF30-dependent and *oxt6-*specific sites are less likely to fall outside of annotated 3’-UTRs than are common sites (Fig 3). Thus, only one CPSF30-dependent site falls within an annotated intron (S3 Fig, panel A); the affected gene is AT4G23670, and the relevant site is a minor site that does not contribute significantly to the transcriptional output of the gene. Four genes possess intronic *oxt6*-specific poly(A) sites (S3 Fig, panel A). None of these sites have the potential to alter the expression of the associated gene. Three genes contain *oxt6*-specific sites within their protein-coding regions (S3 Fig, panel B). These CDS sites are located near the translation termination codons, are relatively minor, and do not significantly impact the overall expression of the respective genes. Interestingly, no CPSF30-dependent sites are found within annotated protein-coding regions.

### Associations of CPSF30 -dependent and -specific sites

**Fig 4:**
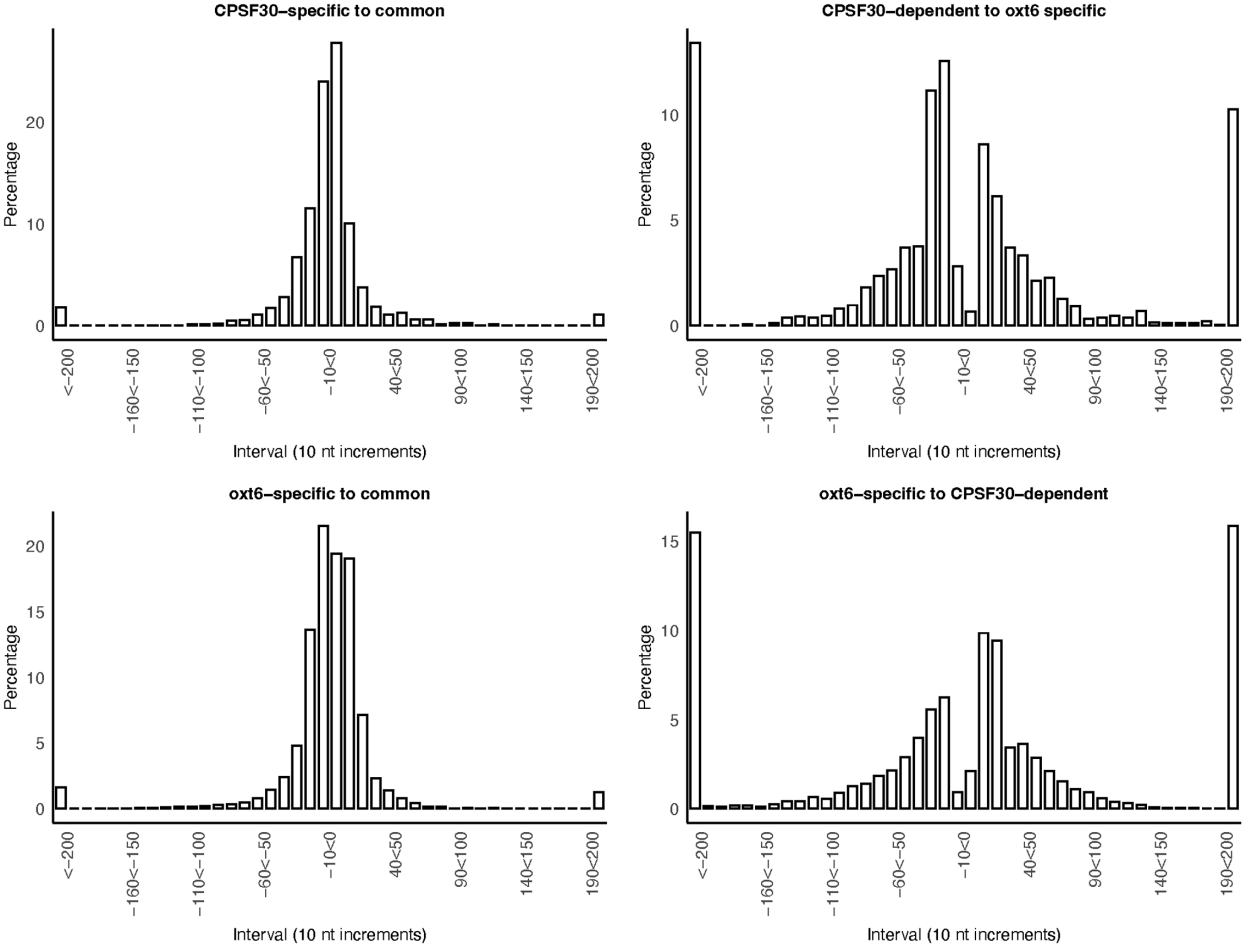
Distances of different classes of sites to other sites. A. Distances from CPSF30-dependent sites to the nearest common site. B. Distances from *oxt6-*specific sites to the nearest common site. C. Distances from CPSF30-dependent sites to the nearest *oxt6-*specific site. D. Distances from *oxt6-*specific sites to the nearest common site. Y-axes denote the percentage of all sites that map to the intervals denoted on the X-axes. X-axes denote ten nt intervals; to make the axis labels more legible, every fifth interval is labeled. The left- and right- most values are the cumulative values for distances less than or greater that 200 nts, respectively.

Alternative polyadenylation can remodel mRNAs, leading to the presence of different sets of possible regulatory elements such as microRNA target sites. For CPSF30-mediated APA to play a significant role in the regulation of gene expression, CPSF30-dependent and *oxt6-*specific sites need to be sufficiently distant from other sites (both strain-specific and common) to allow for substantial differences in mRNA composition. To investigate this possibility, we analyzed the distances between CPSF30-dependent and *oxt6-*specific sites. The results show that the vast majority (>90%) of CPSF30-dependent (Fig 4A) and *oxt6-*specific (Fig 4B) sites are located within 50 nts of a nearby common site. Furthermore, more than 70% of the CPSF30-dependent sites are within 50 nts of an *oxt6*-specific site (Fig 4C). The inverse was also true, with ~ 80% of the *oxt6-*specific sites lying within 50 nts of a CPSF30-dependent site (Fig 4D).

**Fig 5:**
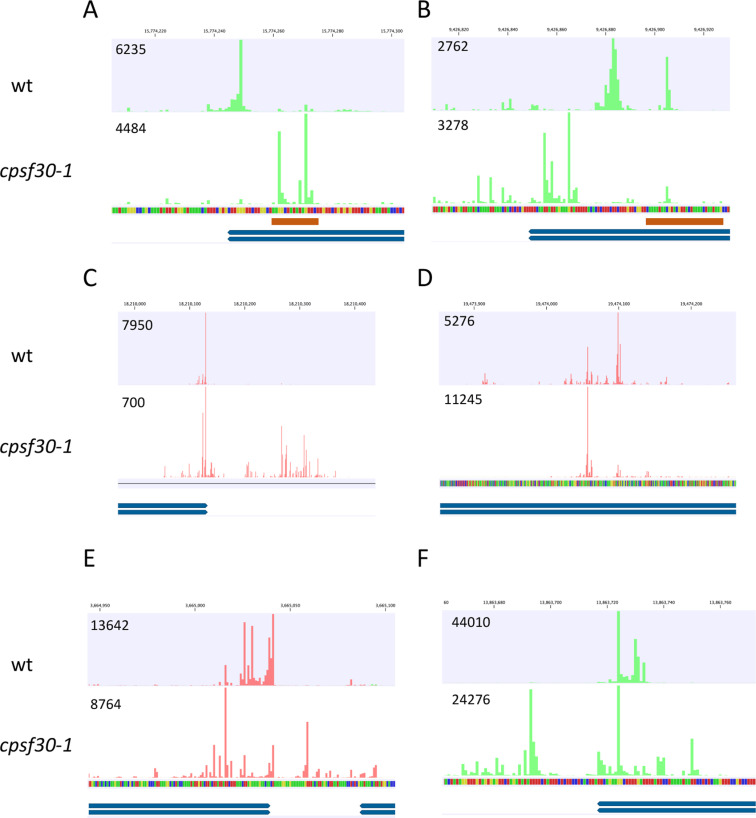
Examples of genes with different classes of sites. For each trace, the upper track depicts mappings of reads derived from sequencing of wild-type samples, the track below this depicts mappings of reads derived from the *cpsf30-1* mutant, and the lower track depicts the gene annotations, respectively. Arrows on the gene annotations (blue bars) indicate the orientation of the gene. Red tics denote reads oriented in the 5’-3’ left to right direction, and green tics denote reads oriented in the 5’-3’ right to left direction. In the sequence tracks, A, G, C, and T are represented by red, yellow, green, and blue symbols, respectively. Note that the scale for Fig 5 C does not permit a nucleotide-level depiction of the sequence. Putative NUEs (A-rich regions) are noted with brown bars beneath the sequence tracks. Numbers at the upper left of each track denote the scale (in read numbers) of each track. Gene identifiers for each panel: A—At2g37600; **B—**At2g22170; **C—**At3g49120; **D—**At2g47450; **E—**At1g10960; **F—**At2g32690.

As suggested by the results in Fig 4, genes with numerous combinations of the three classes of poly(A) sites are possible. Many genes have both CPSF30-dependent and *oxt6-*specific sites (examples shown in Figs 5A and 5B). In some cases, such as shown in Fig. 5A, the *oxt6*-specific sites are in the region that corresponds to the NUE of the CPSF30-dependent sites. However, there are instances (as seen in Fig 5B) where the *oxt6*-specific sites do not align with the likely NUE associated with the CPSF30-dependent sites. This observation challenges the notion that *oxt6*-specific sites are those where cleavage and polyadenylation occurs at the NUEs of CPSF30-dependent sites.

Other genes have both *oxt6*-specific and common sites (example shown in Fig 5C) or CPSF30-dependent and common sites (example shown in Fig 5D). These combinations indicate that common sites and strain-specific sites are not mutually-exclusive, and thus that use of strain-specific sites does not preclude the use of common sites. As indicated in Fig 4, strain-specific sites may be upstream (as seen in Fig 5D) or downstream (as seen in Fig 5C and Fig 5D) of common sites.

Many genes exhibited combinations of common, CPSF30-dependent, and *oxt6*-specific sites (examples shown in Fig 5E and Fig 5F). Numerous configurations were observed, such as instances where common sites were flanked by one or both classes of strain-specific sites (as shown in Fig 5E and Fig 5F), as well as cases where strain-specific sites were positioned on the same sides of common sites (as is seen with some of the common sites in Fig 5E and Fig 5F). Taken together, the results shown in Fig 5 indicate that there is no single “rule” governing the relationships and associations between these three classes of poly(A) sites. In other words, strain-specific sites may lie upstream or downstream of common sites, and also of other strain-specific sites. Moreover, there are no sequence motifs that may define subsets of these sites and arrangements.

APA may alter the ability of microRNAs to target mRNAs. However, only two of the numerous genes with CPSF30-associated mRNA remodeling are associated with possible microRNA targets (S4 Fig).

### Transcripts ending at strain-specific poly(A) sites have slightly lower stabilities

To explore the possible impact of CPSF30-mediated APA on gene expression, we assessed the relative stabilities of mRNA isoforms that terminate at CPSF30-dependent, CPSF30*-*specific, or common poly(A) sites. For this, *Arabidopsis* seedlings were treated with cordycepin for 60 minutes so as to stop transcription, after which RNA was isolated and used to prepare PATSeq libraries. Fractional usages of different poly(A) sites were then calculated on a gene-by-gene basis. If all mRNA isoforms had similar stabilities, then fractional usage should not change upon cordycepin treatment. Unstable mRNA isoforms should show lower fractional usages after cordycepin treatment, and isoforms that are more stable should show higher fractional usages.

**Fig 6:**
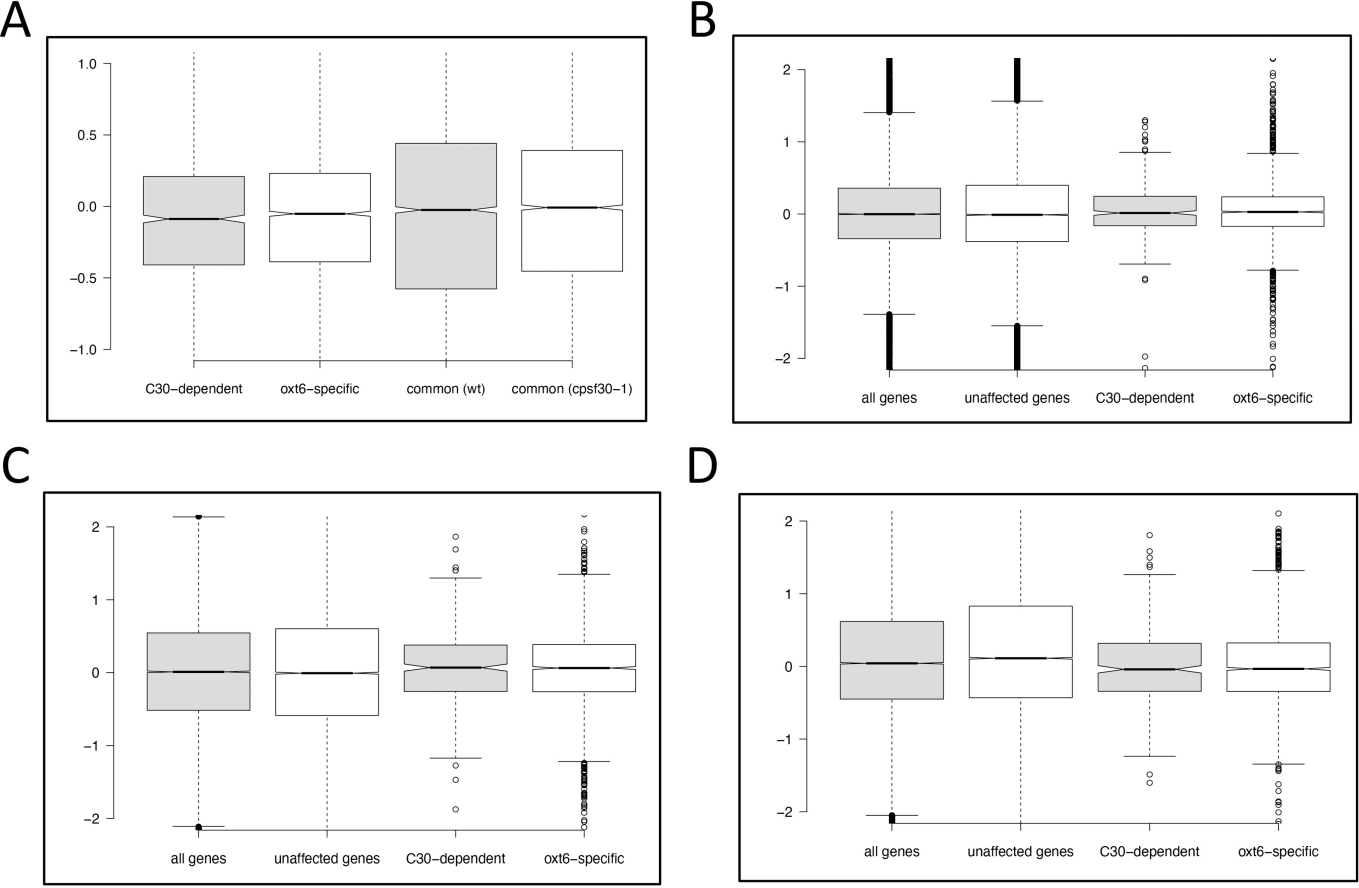
Effects of the *cpsf30-1* mutation on mRNA stability and gene expression. A. Relative stabilities of different classes of mRNAs. “common (wt)”—stabilities of common mRNA isoforms in wt plants; “common (cpsf30-1)” —stabilities of common mRNA isoforms in *cpsf30-1* mutant plants; “C30-dependent”—stabilities of CPSF30-dependent mRNA isoforms in wt plants; “oxt6-specific”—stabilities of *oxt6*-specific mRNA isoforms in the *cpsf30-1* mutant. Y-axis values are log2-transformed values of the relative changes in poly(A) site usage as described in the text. Data for these plots are in S4 File. B. Analysis of changes in gene expression in the *cpsf30-1* mutant compared with wt plants; these results were obtained using data from Yu et al. [23]. C. Analysis of changes in gene expression in the *cpsf30-1* mutant compared with wt plants; these results were obtained using plants grown on defined media as described in the text. D. Analysis of changes in gene expression in the *cpsf30-1* mutant compared with wt plants; these results were obtained using grown in soil as described in the text. In panels B-D, the y-axis values are log2-transformed ratios of the fold-change in expression in the *cpsf30-1* and wt plants. “all genes”—fold-change for all genes; “unaffected genes”—fold-change for genes either lacking strain-specific sites or possessing both CPSF30-dependent and oxt6-specific sites; “C30-dependent”—fold-change for genes with CPSF30-dependent sites; “oxt6-specific”—fold-change for genes with oxt6-specific sites. Data for the plots in panels B, C, and D are in S5 File.

The results of this experiment are summarized in Fig 6A. Compared to the bulk samples from the two genetic backgrounds, the relative stabilities of mRNAs ending at *oxt6*-specific sites were slightly lower, as indicated by the decrease in fractional usage of these sites in cordycepin-treated plants. CPSF30-dependent poly(A) sites also showed slightly lower relative stabilities. In contrast, the fractional usages of common sites remained unchanged after cordycepin treatment.

The slightly lower stabilities of mRNAs ending at strain-specific sites suggests that genes with these sites may show different expression patterns in the wild-type and *cpsf30-1* mutant. Specifically, transcriptional output from genes with CPSF30-dependent sites includes unstable RNAs that are absent in the *cpsf30-1* mutant, while genes with *oxt6-*specific sites produce unstable RNAs that are absent in the wild-type. Genes with both classes of sites may generate unstable RNAs regardless of the genetic background. Therefore, CPSF30-associated APA has the potential to alter overall mRNA levels. To test this, gene expression levels in the wild-type and *cpsf30-1* mutant were compared. For this, differential gene expression was evaluated for each of the individual experiments summarized in Fig 1, rather than pooling the studies as done for identifying CPSF30-dependent poly(A) sites. This approach accounts for the different expression baselines across experiments, which might otherwise obscure CPSF30-related expression changes.

For each experiment, we determined the relative expression of each gene in wild-type and *cpsf30-1* backgrounds. We then plotted the ratios for the complete set of genes, genes with CPSF30-dependent sites, genes with CPS30*-*specific sites, and genes with neither class of sites. The results (Figs 6B–D) reveal little difference in expression across the three gene classes. The expression of genes encoding mRNAs derived from use of strain-specific sites was slightly higher in the two experiments that used plants grown on sterile defined media (Figs 6B and 6C) but slightly lower in plants grown in soil (Fig 6D). This observation aside, the results shown in Figs 6B–D indicate that the expression of genes that encode unstable mRNAs is not significantly different in wt and *cpsf30-1* mutant plants.

## Discussion

### CPSF30, the plant polyadenylation complex, and the specification of the poly(A) site

In mammals and yeast, a large complex recognizes the polyadenylation signal and coordinates subsequent cleavage and polyadenylation of the mRNA. In mammals, the polyadenylation signal (AAUAAA) is bound by two subunits, CPSF30 and WDR33. Prior analyses of poly(A) site usage in the *oxt6* mutant, as well as the results presented in this report, indicate that the plant CPSF30 associates with the A-rich NUE, the presumed counterpart of the mammalian AAUAAA. Specifically, the strong association of AAUAAA-like motifs with CPSF30-dependent sites noted in Yu et al. [23] and seen in this report (Figs 2A and 2B) suggests a direct CPSF30-AAUAAA association.

However, other results presented in this report suggest a more nuanced mechanism for specification of the A-rich NUE. Thus, while there is a decided association of A-rich regions and AAUAAA with CPSF30-dependent poly(A) sites (Figs 2A and 2B), a similar A-rich region is seen in common poly(A) sites (Figs 2E and 2F). It therefore follows that CPSF30 is not necessary for recognition of all A-rich NUEs since usage of the associated common sites is seen in the *oxt6* mutant. This is difficult to reconcile with the means by which AAUAAA is recognized in mammals and yeast. In mammals, the A1 and A2 bases associate with the second zinc finger of CPSF30. Similarly, the first three bases of the yeast polyadenylation signal (itself an A-rich element) associate with the second zinc finger of Yth1 (the yeast ortholog of CPSF30). Thus, in these model systems, the A-rich nature of the polyadenylation signal is determined in large part by CPSF30/Yth1. The fact that an A-rich element in plants can function in the absence of CPSF30 raises interesting questions about the nature of the plant poly(A) complex. Are there proteins that may substitute for CPSF30 in the PAC that may also promote recognition of an A-rich NUE? Can the plant poly(A) complex promote binding to A-rich RNAs without CPSF30 or other RNA-binding subunits?

The features of sites used only in the *oxt6* mutant raise additional questions. The characteristics of these sites suggest an involvement of factors that recognize an apparent U-rich NUE (Figs 2C and 2D). Together, the characteristics of the different classes of poly(A) sites suggest three variants of the plant poly(A) complex: one that includes CPSF30 and shows a strong preference for AAUAAA-like motifs, another where CPSF30 is replaced by different RNA-binding proteins that favor A-rich sequences albeit with a weaker preference for AAUAAA-like motifs, and a third where CPSF30 is substituted with proteins that prefer U-rich sequences. There would be a hierarchy among these complexes, where subunits favoring U-rich NUEs are excluded by those with affinities for A-rich sequences.

### The intersection of CPSF30-mediated APA and gene regulation

In plants, CPSF30 has been linked to various developmental and physiological processes. Mutants lacking CPSF30 expression or activity exhibit altered root and floral organ development [2,15,43], modified responses to biotic [34] and abiotic [18] stresses, altered sensitivity to growth regulators [23,43], and changes in nitrogen-responsive gene expression [1,22]. *Arabidopsis* CPSF30 mutants also exhibit wide-ranging changes in poly(A) site usage (this study [23,32,36,47],). However, unequivocal causal links between specific CPSF30-dependent APA events in target genes and associated phenotypes have yet to be determined. The gene set enrichment analyses (S2 Fig) suggest that APA involving genes associated with plastid and ribosome function may connect CPSF30-associated APA with phenotypic outcomes. However, due to the broad scope of these GO classifications and to the very modest effects of CPSF30-mediated APA on mRNA function, it remains unclear which genes may be directly responsible for CPSF30-associated phenotypes.

In mammals, APA serves to remodel 3’-UTRs in ways that add or subtract regulatory features such as sequences that modulate RNA stability, translation, or localization [1,3]. The distances measured in this report between most CPSF30-dependent sites and common sites are typically less than 30 nts (Fig 4), and none of the numerous genes affected by CPSF30-dependent APA harbor microRNA targets that could conceivably be controlled by CPSF30 (S2 Fig panel A). Moreover, while mRNA isoforms derived from strain-specific poly(A) sites are slightly less stable than other mRNA isoforms (Fig 6A), this subtle difference does not appear to affect the overall expression of the associated genes (Figs 6B–D). Thus, it would seem that CPSF30 does not act broadly through these modes of regulation.

In plants, APA can re-direct transcriptional output into non-productive pathways [53,54]. For example, mRNAs with poly(A) sites situated within protein-coding regions will lack translation termination codons and thus be flagged for degradation via the nonstop decay pathway. Transcripts with 3’ ends that lie within introns may yield mRNAs that are decoded into truncated polypeptides or alternatively may be recognized by the nonsense-mediated decay pathway. However, none of these modes of regulation would appear to be generally applicable to genes affected by CPSF30-dependent APA. Only 8 genes possess strain-specific sites that fall within introns or protein-coding regions (Fig 3, S2 Fig panels A and B). This is a very small fraction of all genes affected by CPSF30-dependent APA. This indicates that CPSF30 probably does not contribute to phenotype by re-directing polyadenylation to non-productive outcomes.

These observations raise questions about the connections between CPSF30-mediated APA and the phenotypes associated with CPSF30 mutants. An earlier review [37] noted that CPSF30-mediated APA had the potential to lead to the production of aberrant proteins, and thus might trigger protein quality-control responses and associated downstream effects. The results presented do not support such a model. Specifically, almost no CPSF30-dependent or *oxt6*-specific sites are located within introns or protein-coding regions, genomic locations associated with the production of aberrant proteins.

The alternative model for CPSF30 function suggests that while CPSF30 subtly affects a large number of genes, it dramatically remodels the transcripts of a select few. This study has uncovered a very small number of possible targets (S3 and S4 Figs). Although none of the identified candidates have been linked to CPSF30-associated phenotypes, and their causal roles in phenotypes observed in CPSF30 mutants remain unclear, the study’s narrow focus—limited by the stringent filters used to identify altered poly(A) sites—leaves open the possibility that other, as yet undiscovered genes may be targets for CPSF30-mediated APA and regulation. For example, poly(A) sites associated with genes whose expression is restricted to growth conditions or tissues that are specific for one or two of the three datasets used to derive the high-confidence set of CPSF30-associated poly(A) sites will be absent from the dataset produced in this study. Sites associated with genes whose expression is highly tissue-specific, or seen only in derived conditions (such as in plants subjected to stresses), will be also be absent from the set of sites compiled here. These hypothetical genes and sites would include strategic targets of CPSF30 that connect APA with physiological outcome.

## Summary and conclusions

This study reveals the presence of three distinct classes of poly(A) site in *Arabidopsis* that depend on CPSF30. The features of these classes suggest a degree of novelty and variability in the poly(A) complex in plants when it comes to the means by which pre-mRNAs are handled by the complex. The characteristics of these sites challenge some existing models of the role of CPSF30 in gene regulation. Specifically, the findings suggest that global regulatory roles for CPSF30 are less plausible than roles by which CPSF30 targets a select group of strategic transcripts and genes.

## Supporting Information

S1 File Library statistics. This file provides general statistics for the 30 individual sequencing samples used in this study.
(XLSX)

S2 File Gene expression data for Fig 1. This file has the expression data generated by CLC Genomics Workbench that was used to generate the PCA plots shown in Fig 1. The reads inputs for each mapping are the files described in S1 File.
(XLSX)

S3 File Lists of poly(A) sites identified in this report. This Supplemental File has five sheets that contain data: PAS Master - pvalue filter: This sheet is a list of the 100,381 PAS that exceed the 250 PAT cutoff for inclusion; in other words, only sites that are represented by at least 250 reads in the complete dataset (18 sequence files) are retained. PAS Master - all sites. Common sites. oxt6-specific. C30-dependent sites.
(XLSX)

S4 File Data for Fig 6A. This Supplemental File has six sheets - this sheet and five that contain data. All sheets pertain to the sequencing of samples prepared from wt and *cpsf30-1* mutant plants treated with cordycepin for 0 or 60 minutes as described in the Materials and Methods. The reads for this experiment were processed and poly(A) sites identified as described in Materials and Methods.
(XLSX)

S5 File Data for Figs 6B–D. This file has three sheets. The first sheet (“Legend”) describes the other two sheets. The second sheet (“expression data”) provides summaries of gene expression for the three experiments used to derive poly(A) site information. The third sheet (“data for Fig 6B–D”) extracts the relevant expression metrics for all genes, genes unaffected by the CPSF30 deficit, genes possessing CPSF30-dependent poly(A) sites, and genes possessing oxt6-specific poly(A) sites; all of these classes of genes are defined and listed in S3 File.
(XLSX)

S1 Fig Summaries of the results of motif analyses of CPSF30- dependent, oxt6-specific, and common polyadenylation sites. Motifs were analyzed using the MEME-CHIP tool in Galaxy [55, 56]. The top five most-frequent motifs occurring in each class of sites are shown.
(PDF)

S2 Fig Gene enrichment analysis of sets of genes impacted by CPSF30-associated alternative polyadenylation. Gene enrichment analysis was conducted using ShinyGO 0.8 [59]. For this analysis, the background set of genes were all those that possess at least one poly(A) site (S2 File). Results are plotted using the “Tree” function. In each tree, the FDR-adjusted p-value, GO classification, and GO class description is provided. The sizes of the blue circles on the plot reflect the numbers of genes associated with each term. For genes with only one poly(A) site, the only significant “Cellular Component” term was GO:0140513 nuclear protein-containing complex; the FDR-adjusted p-value for the enrichment was 2.66E-09, and 283 genes (of a total of 945) were present in this list. In all instances, only terms whose FDR-adjusted p-value for enrichment was less that 10-5 were recovered and plotted.
(PDF)

S3 Fig Genes with strain-specific poly(A) sites that fall within introns (Panel A) or protein-coding regions (Panel B). Reads tracks are placed between annotation tracks; annotations depicted are mRNA (green), microRNA targets (no shading), coding regions (yellow), and genes (blue). The files for the reads tracks (“wt reads” and “cpsf30-1 reads”) show the positions corresponding to the 3’ ends of mapped reads; only the 3’ extremity is depicted. Gene designations are given on the left. For each gene, two browser track views are provided; views on the left show the entire respective gene, and those on the right a “close-up” of the relevant regions. For At1G25054 (Panel A), close-up views of two parts of the gene are shown. Chromosome coordinates are shown at the top of each track set. MicroRNA tracks are as shown in S3 Fig and are included in this figure to convey that most instances of APA are not affected by the presence of miRNA targets. - genes with non-canonical poly(A) sites.
(PDF)

S4 Fig Genes with strain-specific poly(A) sites that flank microRNA target sites. Reads tracks are placed between annotation tracks; annotations depicted are mRNA (green), microRNA targets (red tics), coding regions (yellow), and genes (blue). The files for the reads tracks (“wt reads” and “cpsf30-1 reads”) show the positions corresponding to the 3’ ends of mapped reads; only the 3’ extremity is depicted. Gene designations are given on the left. For each gene, two browser track views are provided; views on the left show the entire respective gene, and those on the right a “close-up” of the relevant regions. Chromosome coordinates are shown at the top of each track set.
(PDF)
