# Supplementary figures and images for: Revisiting CPSF30-mediated alternative polyadenylation in Arabidopsis thaliana
Source: PLoS One. 2025 Feb 24;20(2):e0319180. doi: 10.1371/journal.pone.0319180 (PMC11849871; doi:10.1371/journal.pone.0319180)

CPSF30-dependent sites

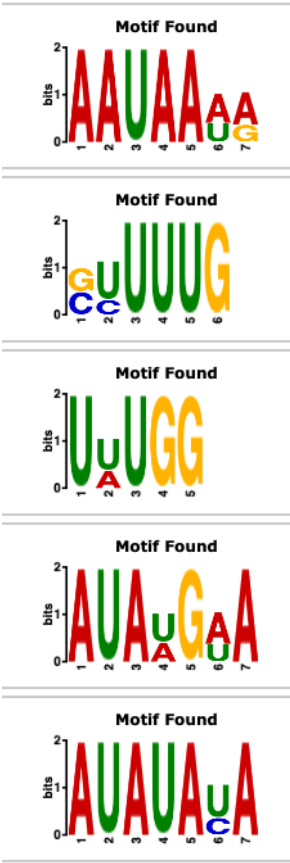

oxt6-specific sites

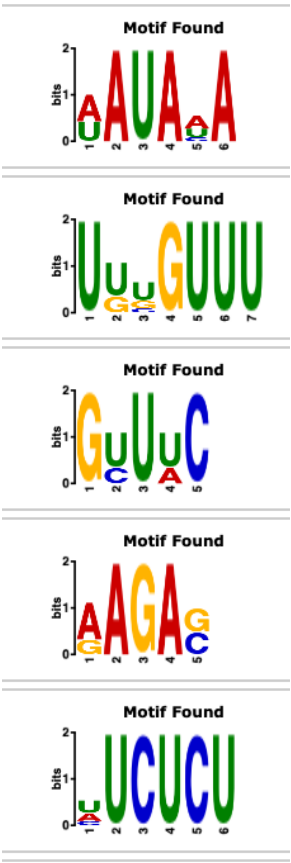

common sites

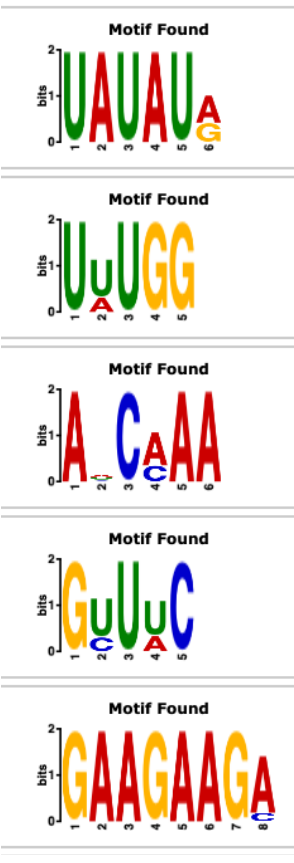

Supplement: S1 Fig — Motifs were analyzed using the MEME-CHIP tool in Galaxy [55, 56]. The top five most-frequent motifs occurring in each class of sites are shown. (PDF) [file pone.0319180.s006.pdf]

A

AT4G23670

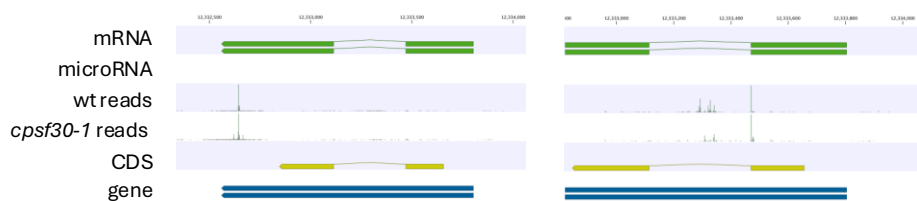

At1G13930

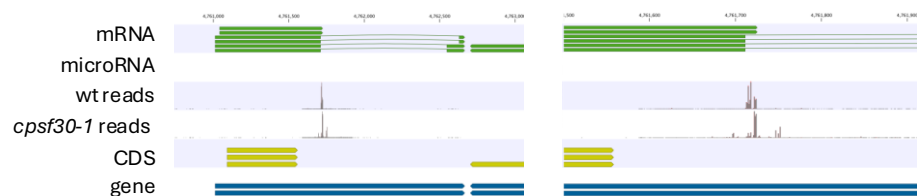

AT4G01380

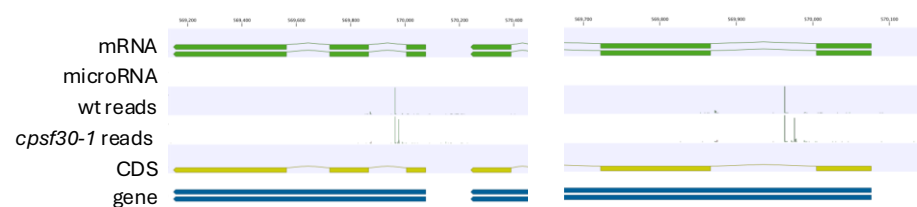

AT3G27030

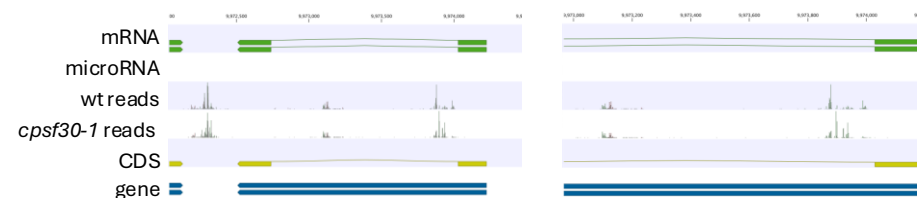

AT1G25054

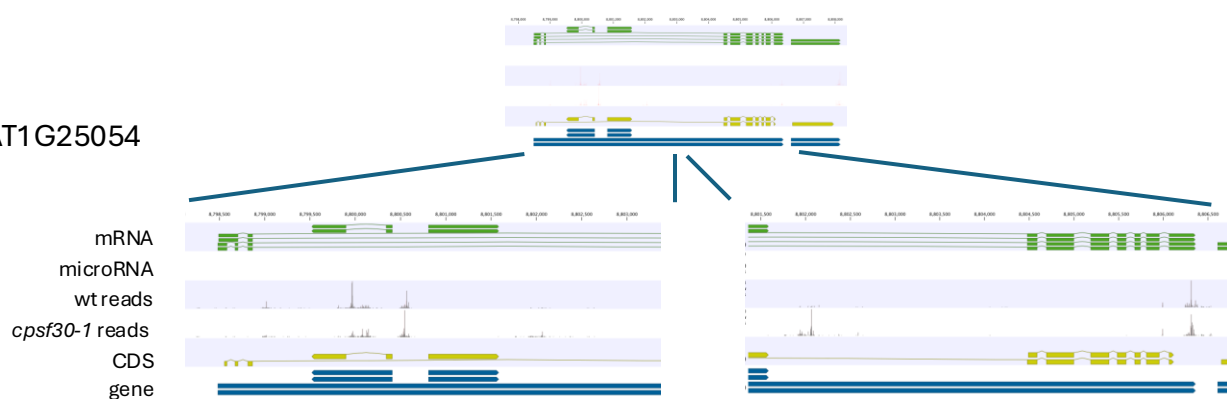

B.

At1G05140

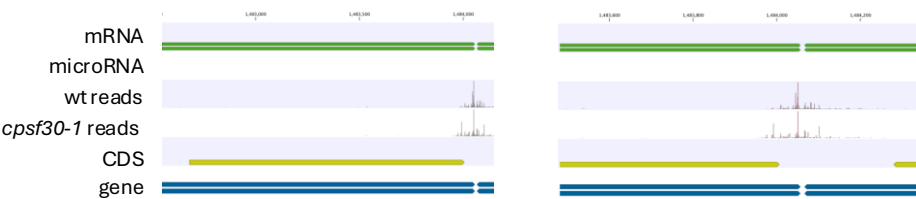

AT3G47836

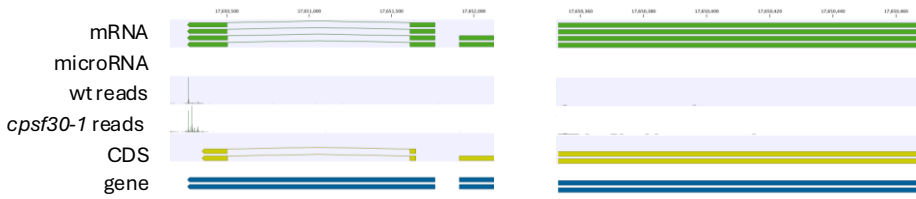

At1G61190

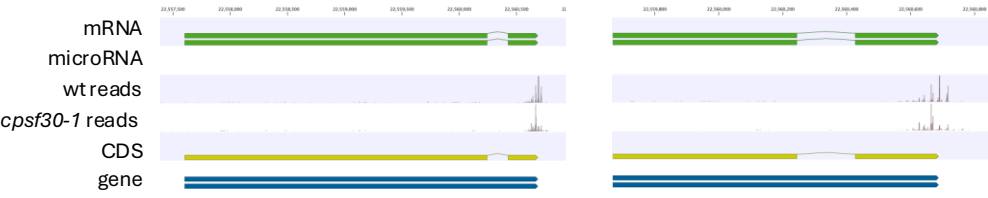

Supplement: S3 Fig — Reads tracks are placed between annotation tracks; annotations depicted are mRNA (green), microRNA targets (no shading), coding regions (yellow), and genes (blue). The files for the reads tracks (“wt reads” and “cpsf30-1 reads”) show the positions corresponding to the 3’ ends of mapped reads; only the 3’ extremity is depicted. Gene designations are given on the left. For each gene, two browser track views are provided; views on the left show the entire respective gene, and those on the right a “close-up” of the relevant regions. For At1G25054 (Panel A), close-up views of two parts of the gene are shown. Chromosome coordinates are shown at the top of each track set. MicroRNA tracks are as shown in S3 Fig and are included in this figure to convey that most instances of APA are not affected by the presence of miRNA targets. - genes with non-canonical poly(A) sites. (PDF) [file pone.0319180.s008.pdf]

AT1G07590

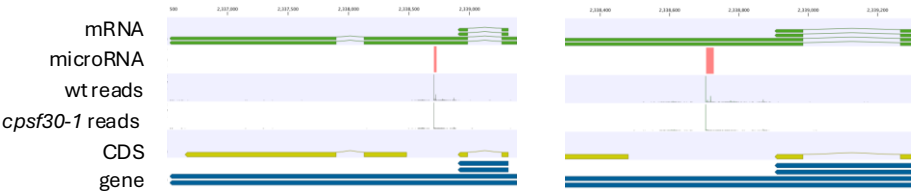

AT1G16240

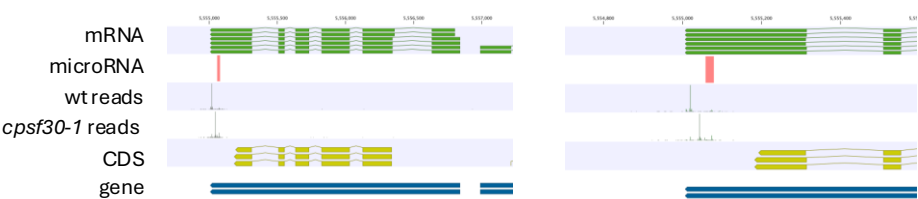

Supplement: S4 Fig — Reads tracks are placed between annotation tracks; annotations depicted are mRNA (green), microRNA targets (red tics), coding regions (yellow), and genes (blue). The files for the reads tracks (“wt reads” and “cpsf30-1 reads”) show the positions corresponding to the 3’ ends of mapped reads; only the 3’ extremity is depicted. Gene designations are given on the left. For each gene, two browser track views are provided; views on the left show the entire respective gene, and those on the right a “close-up” of the relevant regions. Chromosome coordinates are shown at the top of each track set. (PDF) [file pone.0319180.s009.pdf]
