# Supplementary material for: Revisiting CPSF30-mediated alternative polyadenylation in Arabidopsis thaliana
Source: PLoS One. 2025 Feb 24;20(2):e0319180. doi: 10.1371/journal.pone.0319180 (PMC11849871; doi:10.1371/journal.pone.0319180)
Supplement: S2 Fig — Gene enrichment analysis was conducted using ShinyGO 0.8 [59]. For this analysis, the background set of genes were all those that possess at least one poly(A) site (S2 File). Results are plotted using the “Tree” function. In each tree, the FDR-adjusted p-value, GO classification, and GO class description is provided. The sizes of the blue circles on the plot reflect the numbers of genes associated with each term. For genes with only one poly(A) site, the only significant “Cellular Component” term was GO:0140513 nuclear protein-containing complex; the FDR-adjusted p-value for the enrichment was 2.66E-09, and 283 genes (of a total of 945) were present in this list. In all instances, only terms whose FDR-adjusted p-value for enrichment was less that 10-5 were recovered and plotted. (PDF) [file pone.0319180.s007.pdf]

Genes with CPSF30-dependent poly(A) sites

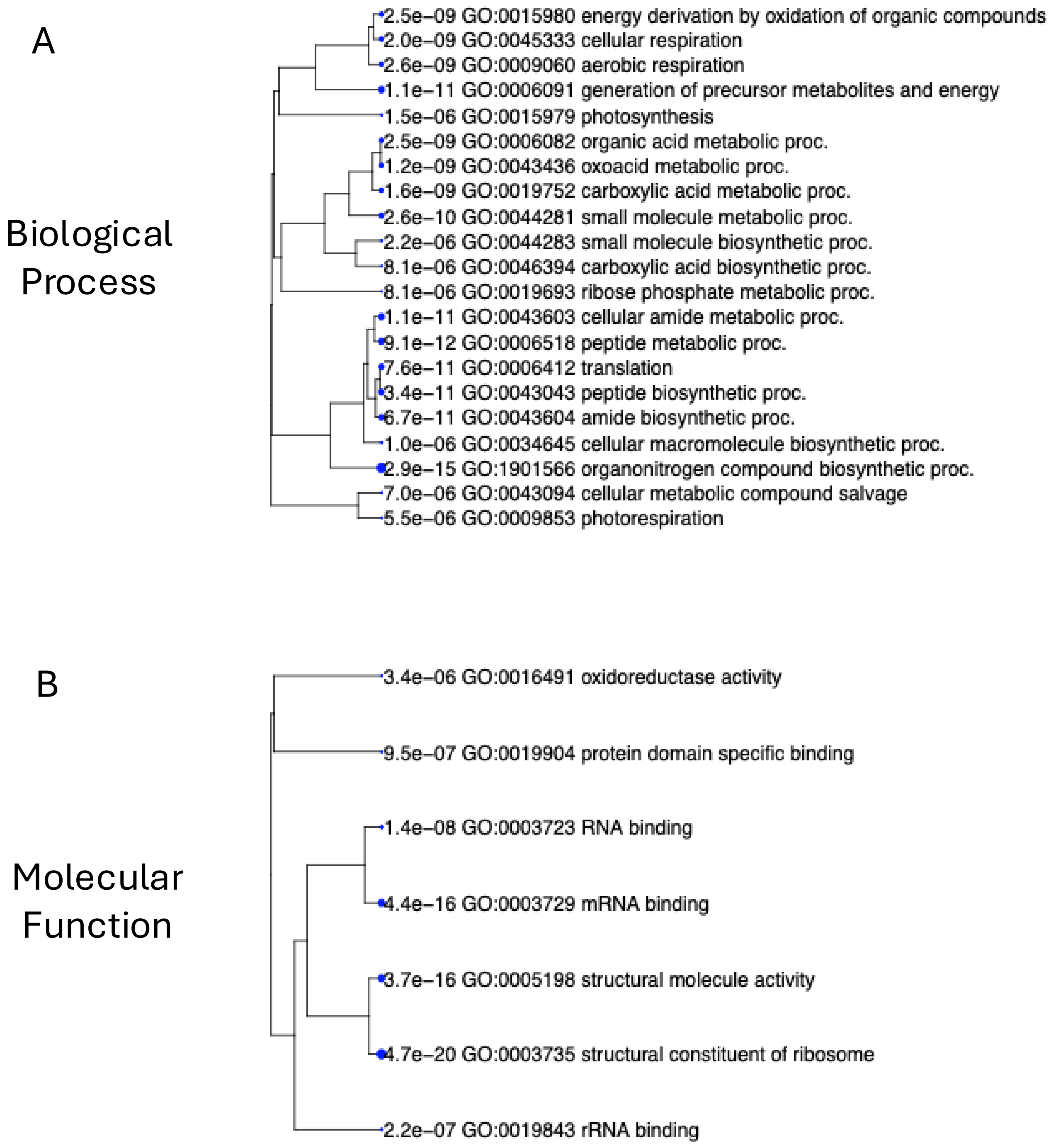

Genes with CPSF30-dependent poly(A) sites

C

Cellular Component

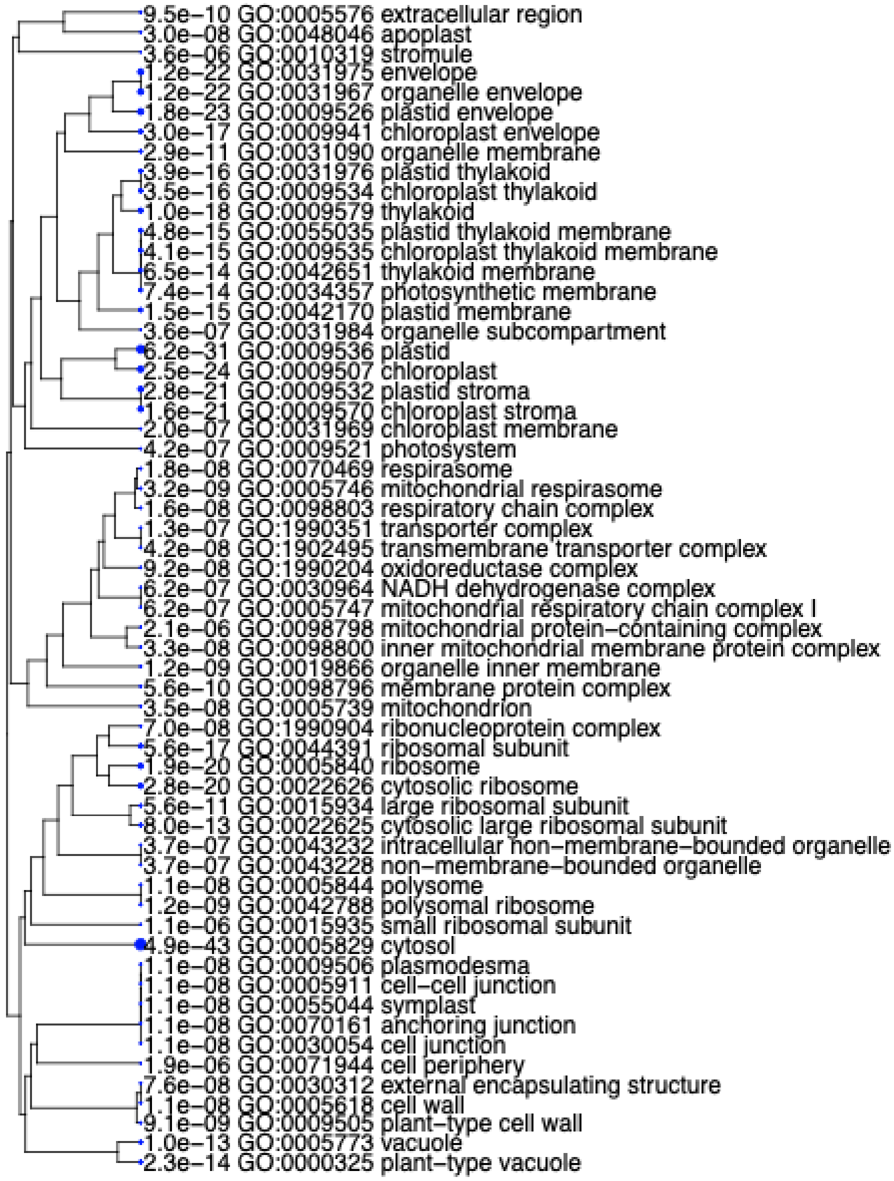

Genes with *oxt6*-specific poly(A) sites

D

Biological Process

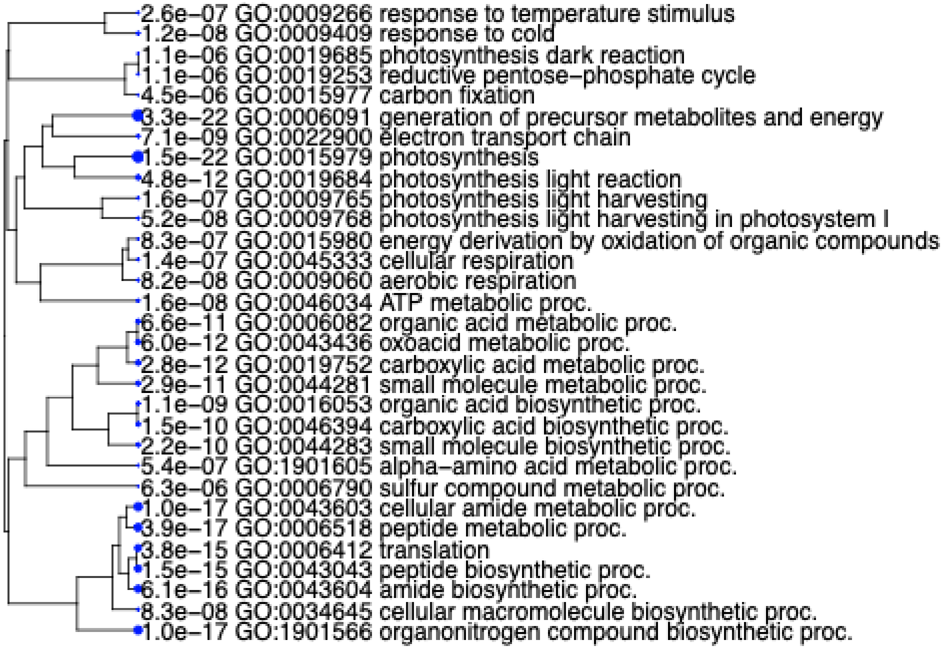


E

Molecular Function

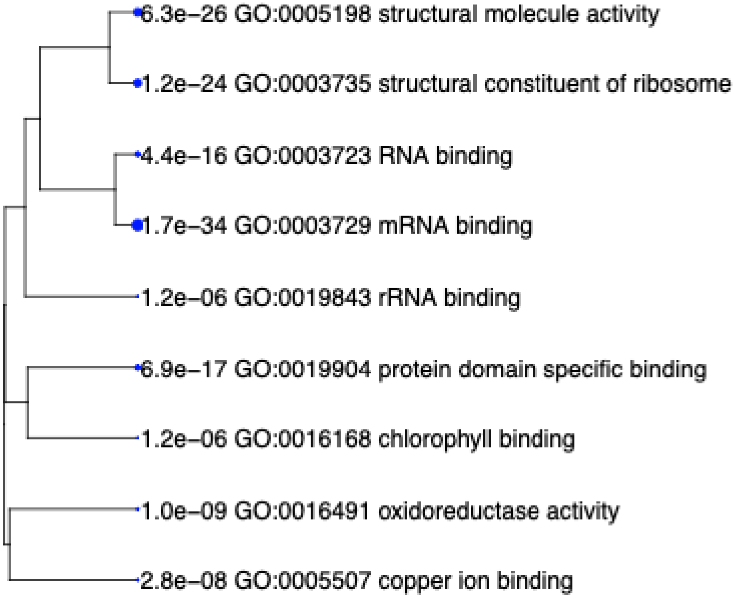

Genes with *oxt6*-specific poly(A) sites

F

Cellular Component

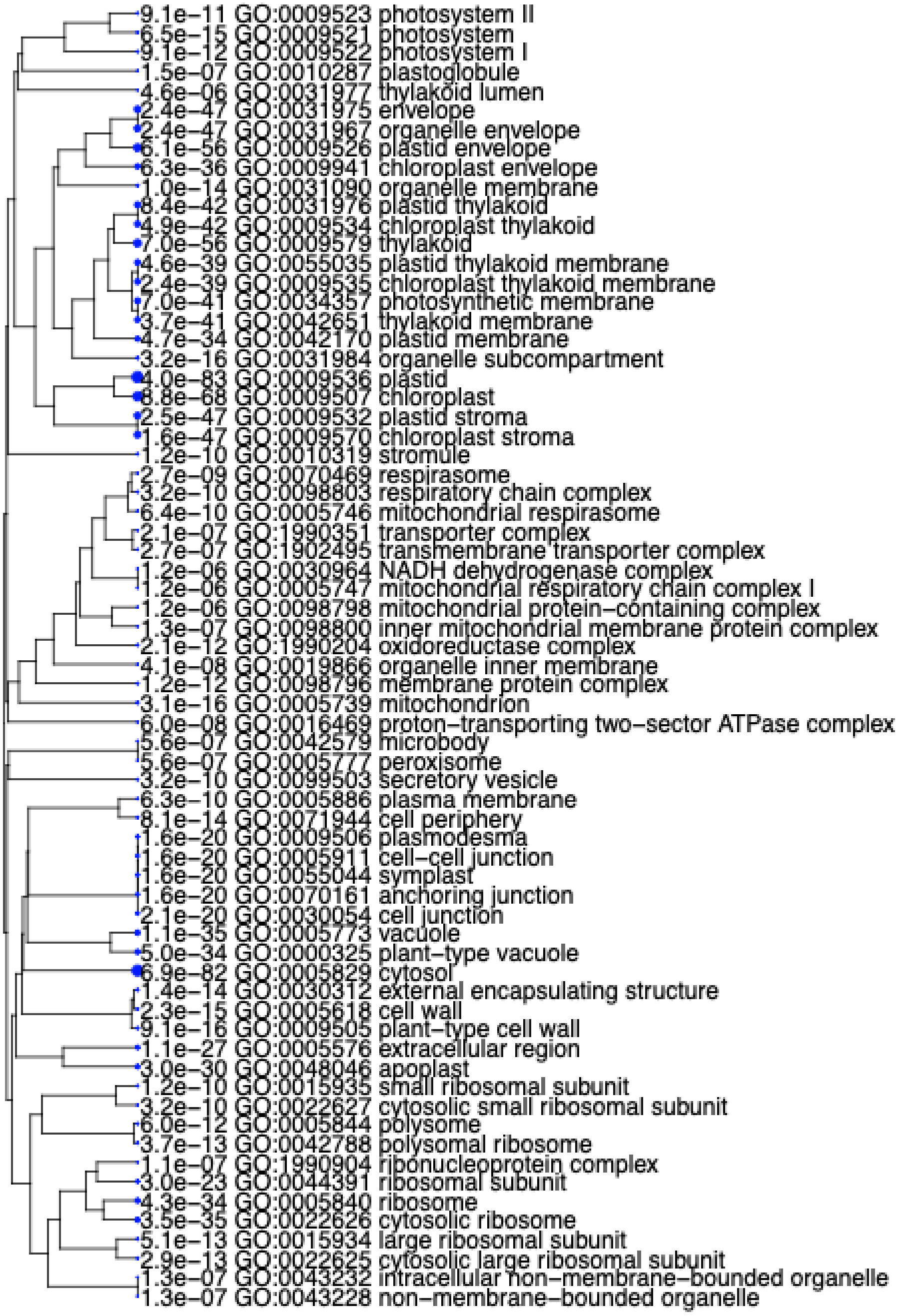

Genes with one PAS

G

Biological Process

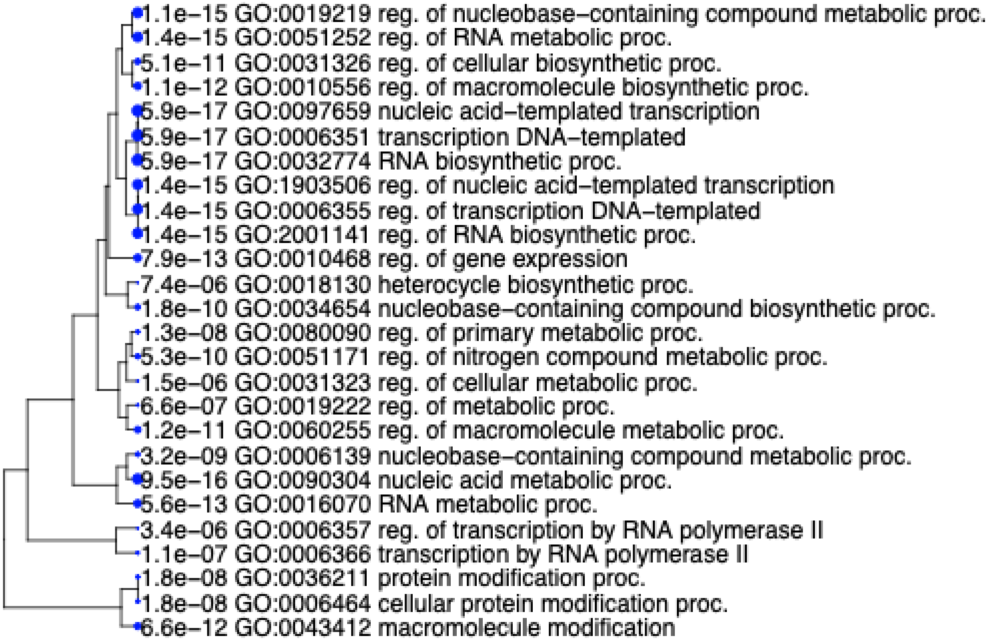


H

Molecular Function

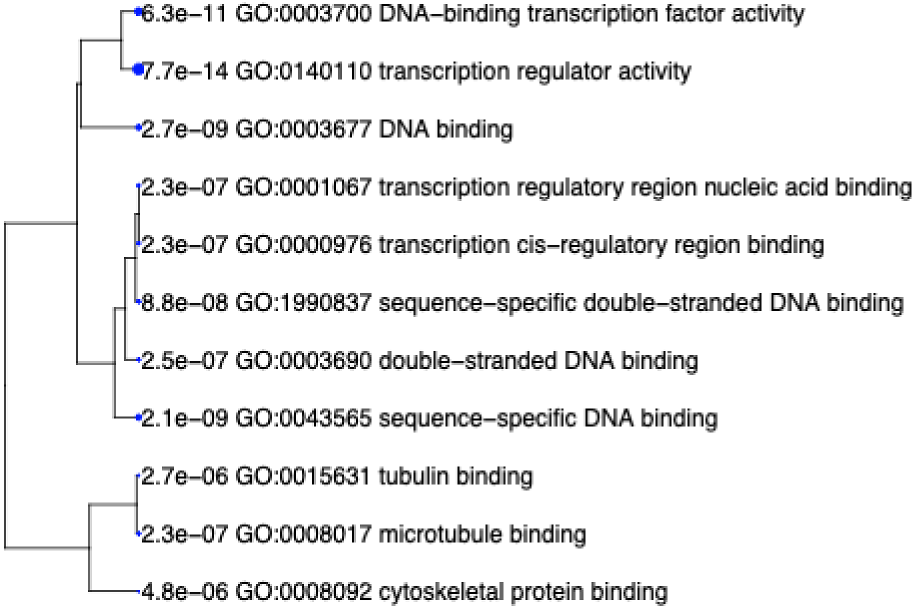


CC – only nuclear genes
